# Supplementary material for: Mapping the Binding Sites of CA125-Specific Antibodies on a Revised Molecular Model of MUC16
Source: Cancers (Basel). 2025 Apr 26;17(9):1458. doi: 10.3390/cancers17091458 (PMC12070838; doi:10.3390/cancers17091458)
Supplement: Supplementary file 1 [file cancers-17-01458-s001.zip › Supplementary_Figures.pdf]

Figure S1

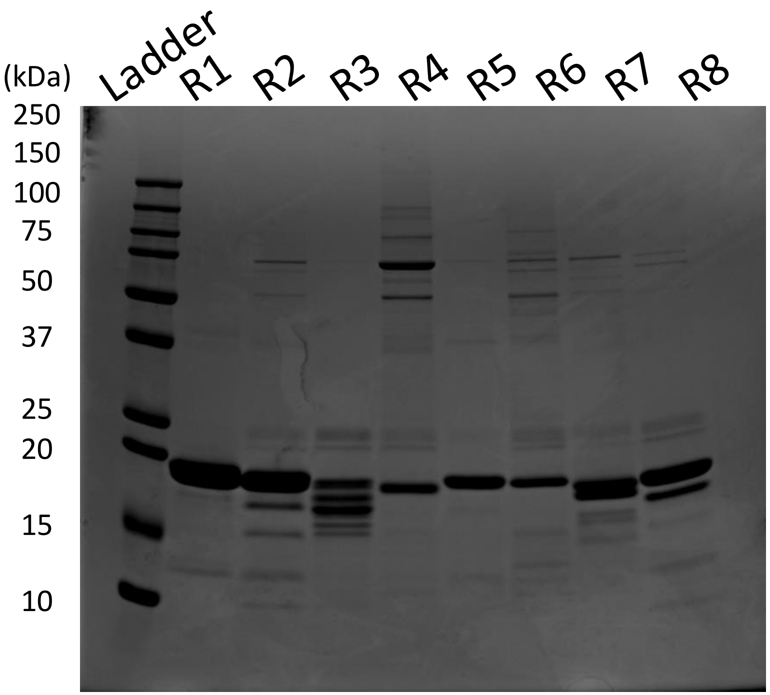
(kDa)
Ladder
R1
R2
R3
R4
R5
R6
R7
R8
250
150
100
75
50
37
25
20
15
10

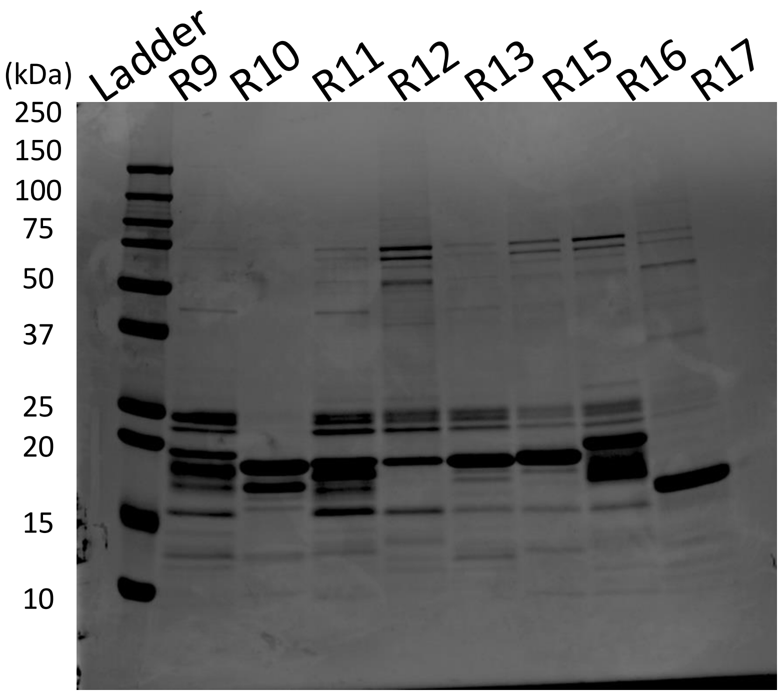
(kDa)
Ladder
R9
R10
R11
R12
R13
R15
R16
R17
250
150
100
75
50
37
25
20
15
10

Figure S2

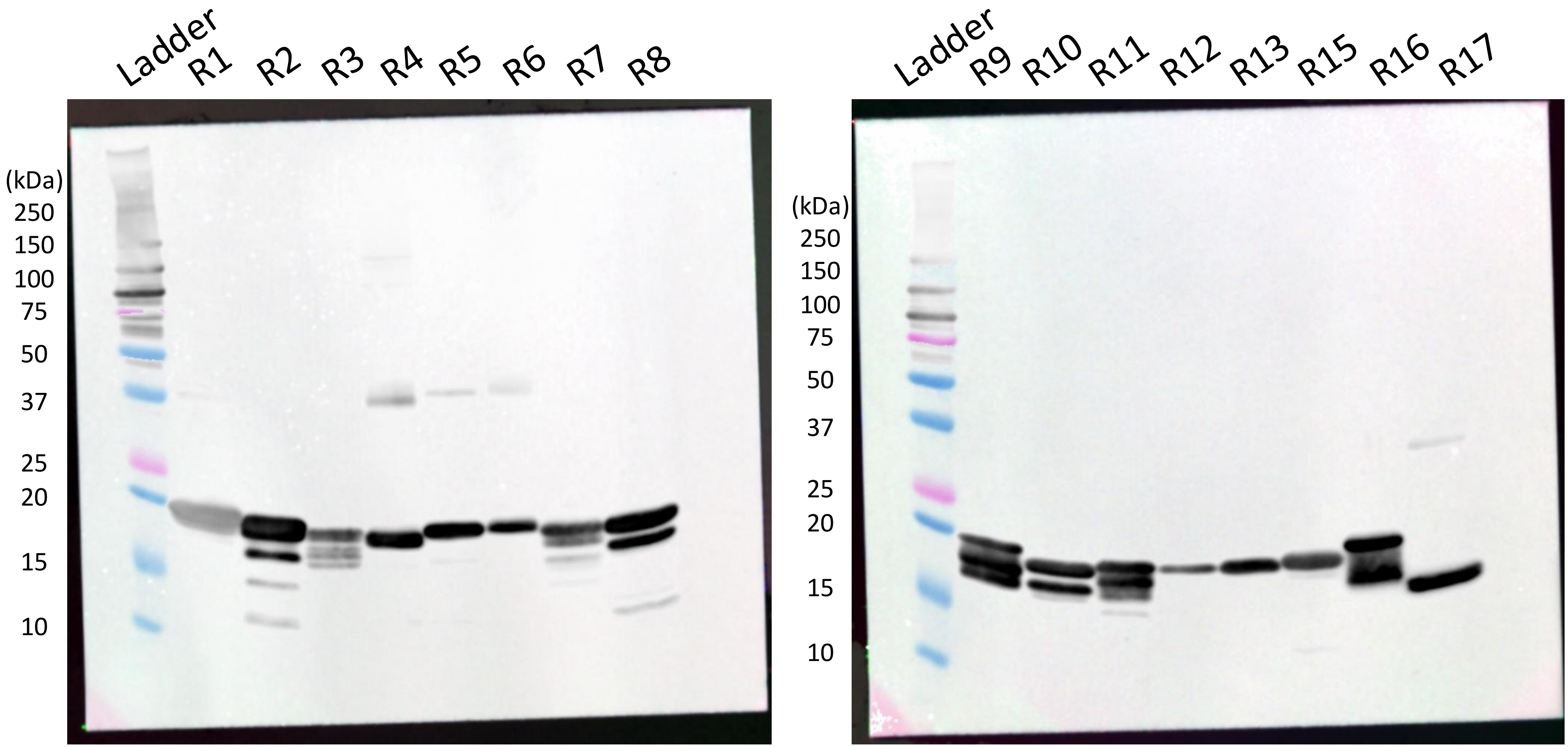
Ladder
R1
R2
R3
R4
R5
R6
R7
R8
(kDa)
250
150
100
75
50
37
25
20
15
10
Ladder
R9
R10
R11
R12
R13
R15
R16
R17
(kDa)
250
150
100
75
50
37
25
20
15
10

Figure S3

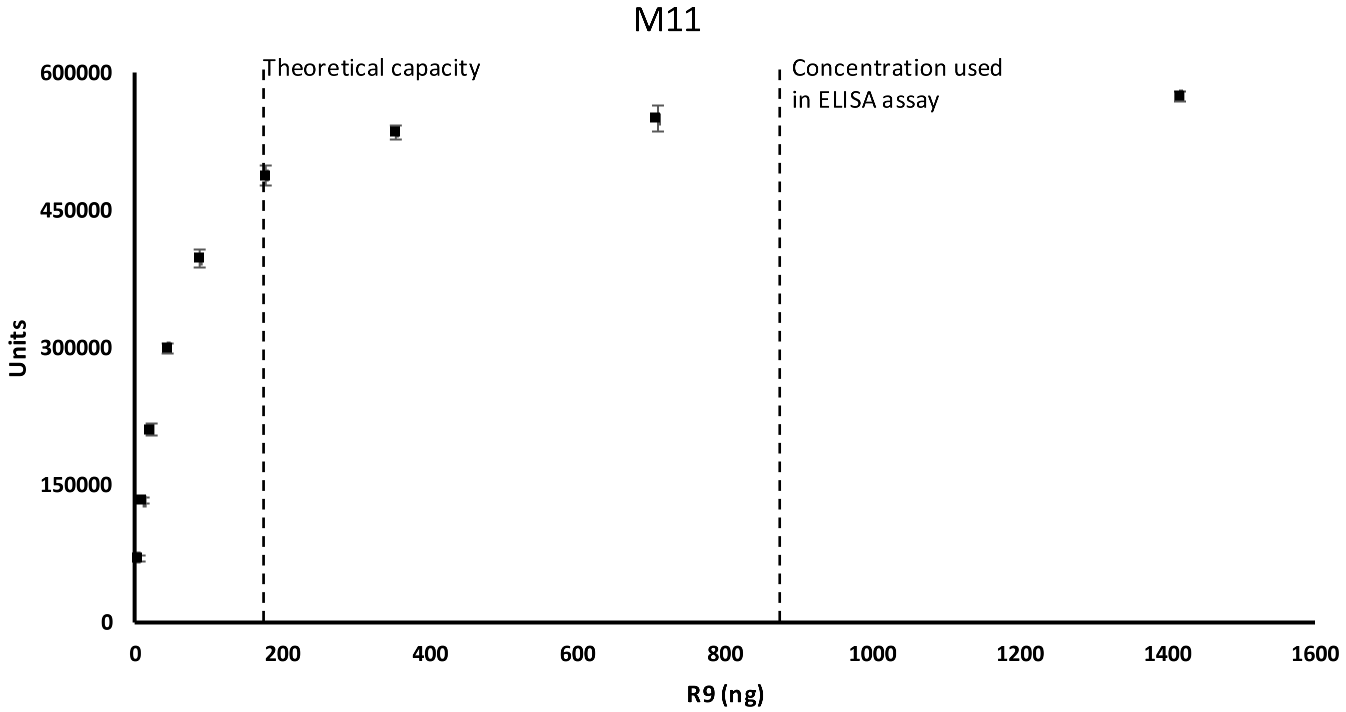
M11
Theoretical capacity
Concentration used
in ELISA assay
600000
450000
300000
150000
0
Units
0
200
400
600
800
1000
1200
1400
1600
R9 (ng)

SI Figure S4

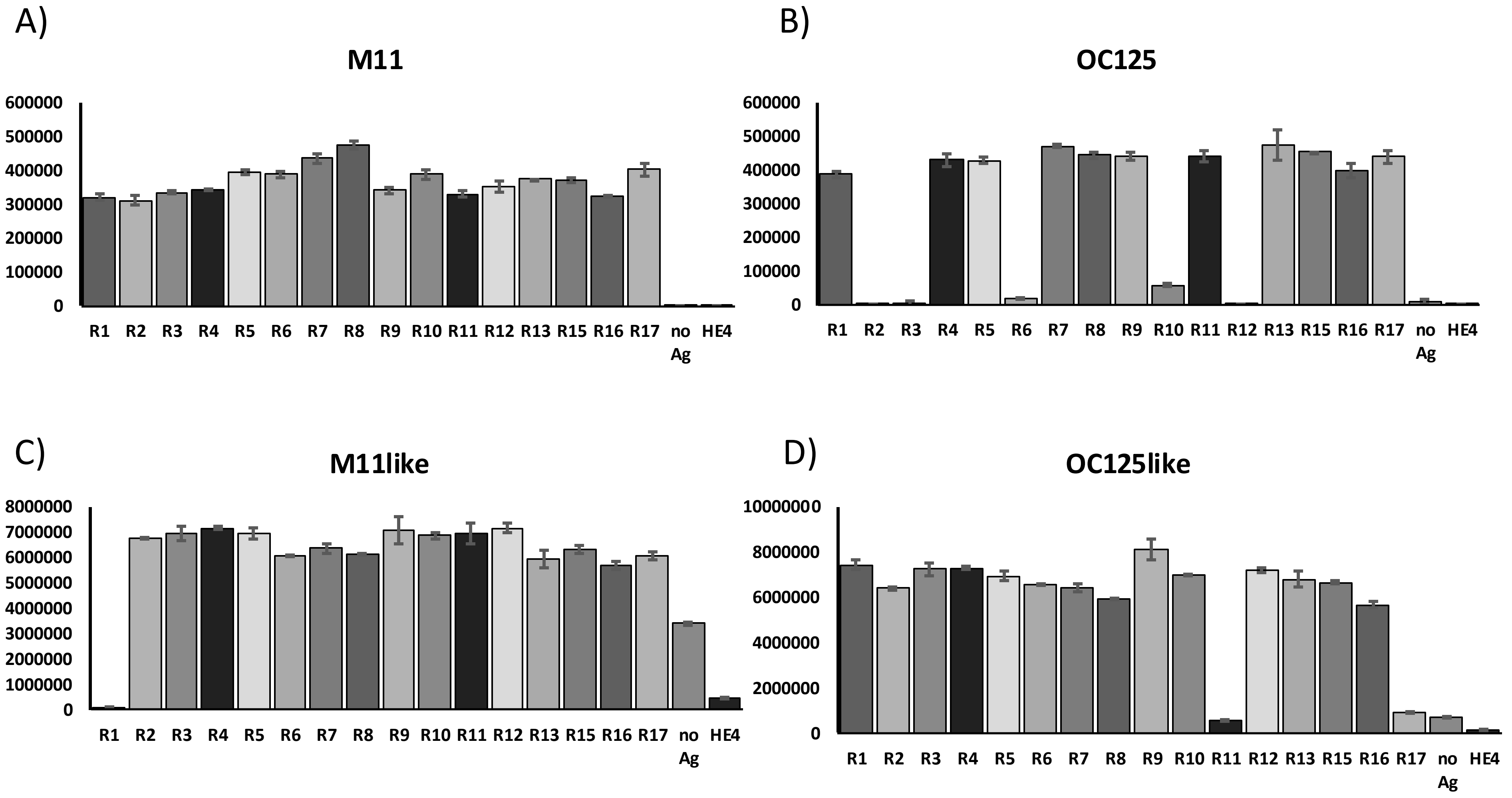
A)
M11
600000
500000
400000
300000
200000
100000
0
R1 R2 R3 R4 R5 R6 R7 R8 R9 R10 R11 R12 R13 R15 R16 R17 no Ag HE4
B)
OC125
600000
500000
400000
300000
200000
100000
0
R1 R2 R3 R4 R5 R6 R7 R8 R9 R10 R11 R12 R13 R15 R16 R17 no Ag HE4
C)
M11like
8000000
7000000
6000000
5000000
4000000
3000000
2000000
1000000
0
R1 R2 R3 R4 R5 R6 R7 R8 R9 R10 R11 R12 R13 R15 R16 R17 no Ag HE4
D)
OC125like
10000000
8000000
6000000
4000000
2000000
0
R1 R2 R3 R4 R5 R6 R7 R8 R9 R10 R11 R12 R13 R15 R16 R17 no Ag HE4

Figure S5

	k_a (1/M*s)			
**Repeat**	**M11**	**OC125**	**M11-like**	**OC125-like**
**R1**	(1.81 ± 0.0) x 10^4	(2.61 ± 0.3) x 10^5	No binding	(3.77 ± 0.2) x 10^5
**R2**	(6.98 ± 2.5) x 10^4	No binding	(1.11 ± 0.0) x 10^5	(8.61 ± 2.5) x 10^4
**R3**	(5.47 ± 3.6) x 10^4	No binding	(2.08 ± 0.7) x 10^5	(2.38 ± 0.2) x 10^5
**R4**	No binding	(2.78 ± 0.9) x 10^5	(2.08 ± 0.3) x 10^5	(8.63 ± 1.3) x 10^5
**R5**	(1.68 ± 0.0) x 10^4	(9.39 ± 0.6) x 10^4	(1.80 ± 0.3) x 10^5	(2.61 ± 0.2) x 10^5
**R6**	No binding	No binding	(2.74 ± 0.6) x 10^5	(2.32 ± 1.2) x 10^5
**R7**	(1.93 ± 0.0) x 10^4	(5.05 ± 0.1) x 10^5	(4.21 ± 0.1) x 10^4	(3.92 ± 0.6) x 10^5
**R8**	(2.07 ± 0.0) x 10^4	(1.10 ± 0.1) x 10^5	(6.46 ± 1.8) x 10^4	(1.12 ± 0.4) x 10^5
**R9**	No binding	NA	(3.03 ± 0.6) x 10^5	(5.23 ± 1.3) x 10^5
**R10**	(1.41 ± 0.0) x 10^4	No binding	(4.37 ± 0.1) x 10^4	(4.24 ± 0.1) x 10^4
**R11**	(1.70 ± 0.1) x 10^4	(3.23 ± 1.1) x 10^5	(1.18 ± 0.3) x 10^5	No binding
**R12**	No binding	No binding	(1.45 ± 1.2) x 10^5	(4.23 ± 0.6) x 10^5
**R13**	(1.48 ± 0.5) x 10^4	NA	(1.91 ± 0.2) x 10^5	(4.66 ± 0.1) x 10^5
**R15**	(1.93 ± 0.0) x 10^4	(1.00 ± 0.0) x 10^5	(3.83 ± 0.2) x 10^4	(1.16 ± 0.2) x 10^5
**R16**	(1.07 ± 0.0) x 10^4	(1.00 ± 0.0) x 10^5	(1.03 ± 0.0) x 10^5	(9.84 ± 1.5) x 10^4
**R17**	(1.70 ± 0.0) x 10^4	(1.00 ± 0.0) x 10^5	(4.04 ± 0.1) x 10^4	No binding

Figure S6

	k_d (1/s)			
Repeat	M11	OC125	M11-like	OC125-like
R1	$(2.91 \pm 0.5) \times 10^{-4}$	$(2.31 \pm 0.3) \times 10^{-4}$	No binding	$(4.79 \pm 0.1) \times 10^{-4}$
R2	1.00×10^{-6}	No binding	$(3.47 \pm 2.47) \times 10^{-6}$	$(4.53 \pm 0.4) \times 10^{-4}$
R3	1.00×10^{-6}	No binding	$(1.26 \pm 0.4) \times 10^{-4}$	$(8.75 \pm 2.6) \times 10^{-5}$
R4	No binding	1.00×10^{-6}	$(2.10 \pm 0.1) \times 10^{-4}$	$(4.38 \pm 0.5) \times 10^{-4}$
R5	1.00×10^{-6}	1.00×10^{-6}	$(3.85 \pm 0.8) \times 10^{-4}$	$(3.61 \pm 0.4) \times 10^{-5}$
R6	No binding	No binding	$(3.64 \pm 0.7) \times 10^{-4}$	$(1.23 \pm 0.4) \times 10^{-4}$
R7	1.00×10^{-6}	$(1.50 \pm 1.4) \times 10^{-5}$	$(1.89 \pm 0.1) \times 10^{-4}$	$(1.56 \pm 0.7) \times 10^{-4}$
R8	1.00×10^{-6}	1.00×10^{-6}	$(1.18 \pm 0.1) \times 10^{-4}$	$(6.40 \pm 2.8) \times 10^{-4}$
R9	No binding	NA	1.00×10^{-6}	$(1.86 \pm 0.5) \times 10^{-4}$
R10	$(1.47 \pm 0.1) \times 10^{-4}$	No binding	$(6.68 \pm 0.2) \times 10^{-5}$	$(1.23 \pm 0.4) \times 10^{-3}$
R11	1.00×10^{-6}	1.00×10^{-6}	$(3.51 \pm 0.3) \times 10^{-4}$	No binding
R12	No binding	No binding	$(2.39 \pm 0.5) \times 10^{-4}$	$(6.95 \pm 2.5) \times 10^{-5}$
R13	$(1.23 \pm 0.2) \times 10^{-6}$	NA	$(3.31 \pm 2.6) \times 10^{-5}$	$(1.32 \pm 0.3) \times 10^{-6}$
R15	1.00×10^{-6}	1.00×10^{-6}	$(1.88 \pm 0.1) \times 10^{-4}$	$(5.68 \pm 1.5) \times 10^{-5}$
R16	$(1.75 \pm 0.9) \times 10^{-4}$	1.00×10^{-6}	1.00×10^{-6}	$(2.18 \pm 0.3) \times 10^{-4}$
R17	1.00×10^{-6}	1.00×10^{-6}	$(2.58 \pm 0.1) \times 10^{-4}$	No binding

Figure S7

	K_D (pM)			
**Repeat**	**M11**	**OC125**	**M11-like**	**OC125-like**
**R1**	16200 ± 2750	878 ± 27	No binding	1280 ± 73
**R2**	22 ± 11*	No binding	31 ± 21	5910 ± 1280
**R3**	33 ± 22*	No binding	707 ± 176	358 ± 87
**R4**	No binding	5± 2*	1040 ± 129	521 ± 57
**R5**	59 ± 2*	11 ± 1*	2170 ± 352	141 ± 25
**R6**	No binding	No binding	1340 ± 25	851 ± 620
**R7**	52 ± 0*	31 ± 29	4480 ± 235	382 ± 117
**R8**	48 ± 1*	9 ± 1*	2130 ± 572	13900 ± 11400
**R9**	No binding	NA	4 ± 1*	476 ± 250
**R10**	10400 ± 710	No binding	1530 ± 33	21900 ± 9000
**R11**	59 ± 4*	5 ± 2*	3710 ± 1490	No binding
**R12**	No binding	No binding	5100 ± 4370	161 ± 56
**R13**	134 ± 82	NA	189 ± 152	3 ± 1
**R15**	52 ± 0*	10.0 ± 0.0*	4950 ± 417	567 ± 254
**R16**	16500 ± 8470	10.0 ± 0.0*	10 ± 0*	2430 ± 741
**R17**	59 ± 1*	10.0 ± 0.0*	6400 ± 243	No binding

Table S1

Repeat number	Recombinant protein sequence (with N terminus 6xHis tag)	Coverage by MS
R1	MGSSHHHHHHSSGLVPRGSHMLEATVPFMVPFTLNFTITNLQYEEDMRHPGSRKFNATERELQGLLKPLFRNSSLEYLYSGCRLASLRPEKDSSATAVDAICTHRPDPEDLGLDRERLYWELSNLTNGIQELGPYTLDRNSLYVNGFTHRSSMPTTSTPGTSTVDVGTSGTPSSSPSPT	91%
R2	MGSSHHHHHHSSGLVPRGSHMLETAGPLLMPFTLNFTITNLQYEEDMRRTGSRKFNTMESVLQGLLKPLFKNTSVGPLYSGCRLTLLRPEKDGAATGVDAICTHRLDPKSPGLNREQLYWELSKLTNDIEELGPYTLDRNSLYVNGFTHQSSVSTTSTPGTSTVDLRTSGTPSSLSSPTIM	92%
R3	MGSSHHHHHHSSGLVPRGSHMLEAAGPLLVPFTLNFTITNLQYGEDMGHPGSRKFNTTERVLQGLLGPIFKNTSVGPLYSGCRLTSLRSEKDGAATGVDAICIHHLDPKSPGLNRERLYWELSQLTNGIKELGPYTLDRNSLYVNGFTHRTSVPTTSTPGTSTVDLGTSGTPFSLPSPA	97%
R4	MGSSHHHHHHSSGLVPRGSHMLETAGPLLVLFTLNFTITNLKYEEDMHRPGSRKFNTTERVLQTLLGPMFKNTSVGLLYSGCRLTLLRSEKDGAATGVDAICTHRLDPKSPGVDREQLYWELSQLTNGIKELGPYTLDRNSLYVNGFTHWIPVPTSSTPGTSTVDLGSGTPSSLPSPT	90%
R5	MGSSHHHHHHSSGLVPRGSHMLETAGPLLVPFTLNFTITNLKYEEDMHCPGSRKFNTTERVLQSLLGPMFKNTSVGPLYSGCRLTLLRSEKDGAATGVDAICTHRLDPKSPGVDREQLYWELSQLTNGIKELGPYTLDRNSLYVNGFTHQTSAPNTSTPGTSTVDLGTSGTPSSLPSPT	100%
R6	MGSSHHHHHHSSGLVPRGSHMLESAGPLLVPFTLNFTITNLQYEEDMHHPGSRKFNTTERVLQGLLGPMFKNTSVGLLYSGCRLTLLRPEKNGAATGMDAICSHRLDPKSPGLNREQLYWELSQLTHGIKELGPYTLDRNSLYVNGFTHRSSVAPTSTPGTSTVDLGTSGTPSSLPSPT	87%
R7	MGSSHHHHHHSSGLVPRGSHMLETAVPLLVPFTLNFTITNLQYGEDMRHPGSRKFNTTERVLQGLLGPLFKNSSVGPLYSGCRLISLRSEKDGAATGVDAICTHHLNPQSPGLDREQLYWQLSQMTNGIKELGPYTLDRNSLYVNGFTHRSSGLTTSTPWTSTVDLGTSGTPSPVPSPT	100%
R8	MGSSHHHHHHSSGLVPRGSHMLETAGPLLVPFTLNFTITNLQYEEDMHRPGSRKFNTTERVLQGLLSPIFKNSSVGPLYSGCRLTSLRPEKDGAATGMDAVCLYHPNPKRPGLDREQLYWELSQLTHNITELGPYSLDRDSLYVNGFTHQNSVPTTSTPGTSTVYWATTGTPSSFPGHT	100%
R9	MGSSHHHHHHSSGLVPRGSHMLEEPGPLLIPFTFNFTITNLHYEENMQHPGSRKFNTTERVLQGLLTPLFKNTSVGPLYSGCRLTLLRPEKHEAATGVDTICTHRVDPIGPGLDRERLYWELSQLTNSITELGPYTLDRDSLYVNGFNPWSSVPTTSTPGTSTVHLATSGTPSSLPGHT	100%
R10	MGSSHHHHHHSSGLVPRGSHMLEAPVPLLIPFTLNFTITNLHYEENMQHPGSRKFNTTERVLQGLLKPLFKSTSVGPLYSGCRLTLLRPEKHGAATGVDAICTLRLDPTGPGLDRERLYWELSQLTNSVTELGPYTLDRDSLYVNGFTHRSSVPTTSIPGTSAVHLETSGTPASLPGHT	100%
R11	MGSSHHHHHHSSGLVPRGSHMLEAPGPLLVPFTLNFTITNLQYEEDMRHPGSRKFNTTERVLQGLLKPLFKSTSVGPLYSGCRLTLLRPEKRGAATGVDTICTHRLDPLNPGLDREQLYWELSKLTRGIIELGPYLLDRGSLYVNGFTHRNFVPITSTPGTSTVHLGTSETPSSLPRPI	91%
R12	MGSSHHHHHHSSGLVPRGSHMLEVPGPLLVPFTLNFTITNLQYEEAMRHPGSRKFNTTERVLQGLLRPLFKNTSIGPLYSSCRLTLLRPEKDKAATRVDAICTHHPDPQSPGLNREQLYWELSQLTHGITELGPYTLDRDSLYVDGFTHWSPIPTTSTPGTSIVNLGTSGIPPSLPETT	100%
R13	MGSSHHHHHHSSGLVPRGSHMLEATGPLLVPFTLNFTITNLQYEENMGHPGSRKFNITESVLQGLLKPLFKSTSVGPLYSGCRLTLLRPEKDGVATRVDAICTHRPDPKIPGLDRQQLYWELSQLTHSITELGPYTLDRDSLYVNGFTQRSSVPTTSTPGTFTVQPETSETPSSLPGPT	80%
R14	MGSSHHHHHHSSGLVPRGSHMLEATGPVLLPFTLNFTIINLQYEEDMHRPGSRKFNTTERVLQGLLMPLFKNTSVSSLYSGCRLTLLRPEKDGAATRVDAVCTHRPDPKSPGLDRERLYWKLSQLTHGITELGPYTLDRHSLYVNGFTHQSSMTTTRTPDTSTMHLATSRTPASLSGPT	N/A
R15	MGSSHHHHHHSSGLVPRGSHMLETASPLLVLFTINFTITNLRYEENMHHPGSRKFNTTERVLQGLLRPVFKNTSVGPLYSGCRLTLLRPKKDGAATKVDAICTYRPDPKSPGLDREQLYWELSQLTHSITELGPYTLDRDSLYVNGFTQRSSVPTTSIPGTPTVDLGTSGTPVSKPGPS	99%
R16	MGSSHHHHHHSSGLVPRGSHMLEAASPLLVLFTLNFTITNLRYEENMQHPGSRKFNTTERVLQGLLRSLFKSTSVGPLYSGCRLTLLRPEKDGTATGVDAICTHHPDPKSPRLDREQLYWELSQLTHNITELGPYALDNDSLFVNGFTHRSSVSTTSTPGTPTVYLGASKTPASIFGPS	76%
R17	MGSSHHHHHHSSGLVPRGSHMLEAASHLLILFTLNFTITNLRYEENMWPGSRKFNTTERVLQGLLRPLFKNTSVGPLYSGCRLTLLRPEKDGEATGVDAICTHRPDPTGPGLDREQLYLELSQLTHSITELGPYTLDRDSLYVNGFTHRSSVPTTSTGVVSEE	100%
R18	MGSSHHHHHHSSGLVPRGSHMLEPFTLNFTINNLRYMADMGQPGSLKFNITDNVMQHLLSPLFQRSSLGARYTGCRVIALRSVKNGAETRVDLLCTYLQPLSGPGLPIKQVFHELSQQTHGITRLGPYSLDKDSLYLNGYNEPGPDEPPTTPKPATTFLPPLSEATT	N/A
R19	MGSSHHHHHHSSGLVPRGSHMLEAMGYHLKTLTLNFTISNLQYSPDMGKGSATFNSTEGVLQHLLRPLFQKSSMGPFYLGCQLISLRPEKDGAATGVDTTCTYHPDPVGPGLDIQQLYWELSQLTHGVTQLGFYVLDRDSLFINGYAPQNLSIRGEYQINFHIVNWNLSNPDPT	N/A

Table S2

Repeat	Ratio
R1	0.94
R2	0.64
R3	0.35
R4	0.44
R5	0.92
R6	0.54
R7	0.80
R8	0.57
R9	0.27
R10	0.51
R11	0.38
R12	0.25
R13	0.50
R15	0.58
R16	0.45
R17	0.75

Table S3

Repeat	Ratio
R1	1.00
R2	0.70
R3	0.58
R4	0.85
R5	0.96
R6	0.93
R7	0.92
R8	0.66
R9	0.28
R10	0.54
R11	0.36
R12	1.00
R13	1.00
R15	1.00
R16	0.41
R17	0.90

Document S1 (attached Excel file)
